# Biofunctionalized Lysophosphatidic Acid/Silk Fibroin Film for Cornea Endothelial Cell Regeneration

**DOI:** 10.3390/nano8050290

**Published:** 2018-04-30

**Authors:** Joo Hee Choi, Hayan Jeon, Jeong Eun Song, Joaquim Miguel Oliveira, Rui Luis Reis, Gilson Khang

**Affiliations:** 1 Department of BIN Convergence Technology, Deokjin-gu, Jeonju-si, Jeollabuk-do 54896, Korea; zooheechoi@jbnu.ac.kr (J.H.C.); wjsgkdis@hanmail.net (H.J.); songje@jbnu.ac.kr (J.E.S.); 2 Department of Polymer Nano Science & Technology and Polymer BIN Research Center, Chonbuk National University, Deokjin-gu, Jeonju-si, Jeollabuk-do 54896, Korea; 3 3B’s Research Group—Biomaterials, Biodegradables and Biomimetics, University of Minho, Headquarters of the European Institute of Excellence on Tissue Engineering and Regenerative Medicine, AvePark—Parque de Ciência e Tecnologia, Zona Industrial de Gandra, 4805-017 Barco, Guimarães, Portugal; miguel.oliveira@dep.uminho.pt (J.M.O.); rgreis@dep.uminho.pt (R.L.R.); 4 ICVS/3B’s—PT Government Associated Laboratory, Braga/Guimarães, Portugal; The Discoveries Centre for Regenerative and Precision Medicine, Headquarters at University of Minho, Avepark, 4805-017 Barco, Guimarães, Portugal

**Keywords:** cornea endothelial cells, tissue engineering, regeneration, silk fibroin, lysophosphatidic acid

## Abstract

Cornea endothelial cells (CEnCs) tissue engineering is a great challenge to repair diseased or damaged CEnCs and require an appropriate biomaterial to support cell proliferation and differentiation. Biomaterials for CEnCs tissue engineering require biocompatibility, tunable biodegradability, transparency, and suitable mechanical properties. Silk fibroin-based film (SF) is known to meet these factors, but construction of functionalized graft for bioengineering of cornea is still a challenge. Herein, lysophosphatidic acid (LPA) is used to maintain and increase the specific function of CEnCs. The LPA and SF composite film (LPA/SF) was fabricated in this study. Mechanical properties and in vitro studies were performed using a rabbit model to demonstrate the characters of LPA/SF. ATR-FTIR was characterized to identify chemical composition of the films. The morphological and physical properties were performed by SEM, AFM, transparency, and contact angle. Initial cell density and MTT were performed for adhesion and cell viability in the SF and LPA/SF film. Reverse transcription polymerase chain reactions (RT-PCR) and immunofluorescence were performed to examine gene and protein expression. The results showed that films were designed appropriately for CEnCs delivery. Compared to pristine SF, LPA/SF showed higher biocompatibility, cell viability, and expression of CEnCs specific genes and proteins. These indicate that LPA/SF, a new biomaterial, offers potential benefits for CEnCs tissue engineering for regeneration.

## 1. Introduction

Cornea is the outer layer of the eye and has three individual layers: the epithelium, stroma, and endothelium [1]. The corneal endothelium is a barrier for metabolic activity that plays an important role in maintaining transparency by utilizing an ATPase pump [2]. Damaged or diseased corneal endothelial cells (CEnCs) are difficult to regenerate due to break down of G1-cell cycle phase. Loss of cell density which is caused by expansion of CEnCs cell size rather than proliferation is also a factor in degeneration of CEnCs. A loss of CEnCs result in corneal edema, blindness, visual acuity, etc. [3,4]. There are nearly 10 million cases of worldwide vision loss due to corneal blindness [5,6]. Therefore, cornea transplantation or replacement of the endothelial cell layer is needed [3]. However, it is reported that although the corneal graft rejection rate is less than 10%, the immunological rejection rate increases to 25% after 4–5 years of implantation and continuously increases over time [6,7,8]. Thus, the development of tissue engineering strategies for an effective alternative to conventional corneal grafts is increasing, and new biomaterials are required for the development of corneal replacement [6,7,9,10,11]. The important factors that should be considered for bioengineered corneas are transparency, biodegradability, biocompatibility, water permeability, possession of essential nutrients for CEnCs, and appropriate mechanical properties for ease handling [2].

*Bombyx mori* silk is a structural protein and is widely used in tissue engineering biomaterials due to its biocompatibility, biodegradation, tunable mechanical properties, and non-immunogenic response in vivo [12,13,14,15]. Silk substrates have been shown to support cell adhesion, mobility, proliferation, and differentiation by mimicking extracellular matrix (ECM) [16,17,18,19,20]. The silk material is made in variety forms such as sponge, hydrogel, fiber, and film [16,17,19,20,21,22,23,24,25,26,27]. The most often-used structure for corneal tissue engineering is film [2,3,15,28,29,30,31,32,33]. However, studies are still preceding to improve silk material properties to enhance cell function, proliferation, and biocompatibility [3,12,15,30,32]. Silk fibroin in film form can be incorporated with bio-functional molecules and other biomaterials to generate functional matrices. Also, patterned silk film surfaces can produce high-resolution surface features [21,22,23,24,25,26,27,28,29,30,31,32,33,34,35].

Herein, lysophosphatidic acid-incorporated silk fibroin film (LPA/SF) was designed for an efficient bioengineering of CEnCs graft. LPA is an endogenous glycerophospholipid signaling molecule, a ligand activator, and has been reported to stimulate growth of fibroblasts, keratinocytes, and endothelial cells, and plays many biological functions in the nervous system [36,37]. The LPA is a critical serum component and affects cell attachment, proliferation, migration, and viability [38]. Epithelial cells, fibroblasts, or platelets are reported to release LPA at injured or inflammation sites [38,39,40]. Also, LPA is released in the injured cornea, and it is predicted that these factors support cell migration and proliferation to regenerate a wound [38].

The LPA/SF was characterized both mechanically and biochemically to determine suitability of CEnCs carrier.

## 2. Materials and Methods

### 2.1. Preparation of SF Solution

Silkworm cocoons were cut into the pieces, and 10 g of silkworm cocoons were added into a boiling 0.02 M Na_2_CO_3_ (Showa Chemical, Tokyo, Japan) in distilled water (DW) for 30 min to remove sericin. After 30 min, silkworm cocoons were washed with DW and fully dried in 60 °C oven for overnight. The dried SF was dissolved in a 9.3M LiBr (Kanto chemical, Tokyo, Japan) for 4 h in the 60 °C to prepare SF solution. The LiBr was removed via dialysis of SF solution in a dialysis tube (SnakeSkin^®^Dialysis Tubing 3500 MWCO, Thermo Fisher Scientific, Waltham, MA, USA) for 48 h, and the SF aqueous solution was kept at 4 °C until usage.

### 2.2. Fabrication of LPA/SF

Pure 8% SF aqueous solution was used in this study. The LPA (Sigma-Aldrich, St. Louis, MO, USA) was dissolved in chloroform:methnol:acetic acid 95:5:5 and incorporated in SF aqueous solution to make the final concentration of 20 μM LPA/SF. The SF and LPA/SF solution were transferred to petri dish to make a thickness of 6–8 μm films and dried under a clean bench to avoid contamination. The films were cross-linked with methanol for 30 min at room temperature. For further sterilization, the films were treated with 70% ethanol under a UV light for 30 min and washed 3 times with PBS for 20 min.

### 2.3. Characterizations

The chemical structure of SF and LPA/SF was analyzed using ATR-FTIR (Perkin Elmer, Waltham, MA, USA) at the spectra wavelength range of 4000–400 cm^−1^.

The morphology was investigated by field emission scanning electron microscopy (FESEM, Hitachi S4700) and atomic force microscopy (AFM) on a Scanning Probe Microscope XE 70 (Multimode-8, Bruker, Billerica, MA, USA). Contact angle characterization was carried out by employing water contact goniometer (TantecTM, CAM-PLUS Micro, Schaumburg, IL, USA) to measure the hydrophilicity of tissue culture polystyrene (TCP), SF, and LPA/SF. Transparency of SF and LPA/SF was evaluated by SYNERGY Mx spectrophotometer (BioTek, Winooski, VT, USA) at a wavelength range of 380 nm–780 nm after immersing in PBS.

### 2.4. Isolation and Culture of Rabbit CEnCs (rCEnCs)

rCEnCs were cultured in endothelial growth medium-2 (EGM-2, Lonza, Walkersville, MD, USA) supplemented with 10% fetal bovine serum (FBS, Gibco, Big Cabin, OK, USA) and 1% penicillin/streptomycin (PS, Gibco, USA), epidermal growth factor (EGF), vascular endothelial growth factor (VEGF), fibroblast growth factor (FGF), insulin-like growth factor (IGF), hydrocortisone, gentamicin, and amphotericin-B under standard culture conditions (5% CO_2_, and 37 °C). rCEnCs were isolated from New Zealand white rabbits (4 weeks old, Female). Animal experiment procedures were approved by Chonbuk National University Animal Care Committee, Jeonju, South Korea. Briefly, eyeballs were removed from rabbits and transferred to PBS. The eyeballs were washed 3 times with PBS under a clean bench. Soft tissues were removed, and cornea was cut from the eyeball. The endothelium with Descemet membrane was stripped from stroma. rCEnCs were transferred into a 0.2% collagenase A (Roche, Germany) and digested for 40 min in an incubator with the condition of 5% CO_2_, and 37 °C. After digestion, the solution was centrifuged at 1500 rpm for 5 min at 4 °C. The rCEnCs pallet was suspended in the culture media and incubated under standard conditions (5% CO_2_, and 37 °C). The media was changed every 2 days, and passage 0 of primary rCEnCs was used for this study.

### 2.5. Morphology Analysis

The rCEnCs were seeded on the SF and LPA/SF at density of 100 cells/mm^2^ and cultured in EGM-2 medium for 5 days. The culture media was changed every 2 days. The media solution was removed, and the films were washed with PBS. The films with adhered cells were fixed with 2.5% glutaraldehyde (Sigma-Aldrich, USA) and different concentrations of ethanol (50%, 60%, 70%, 80%, 90%, and 100%) were used sequentially to dehydrate the films. The ethanol was changed every 20 min for dehydration and dried for 24 h in room temperature before FESEM evaluation.

### 2.6. Initial Attachment

The density of 500 cells/mm^2^ was seeded on TCP, SF, and LPA/SF in the culture media. After 30 min of culture, the medium was aspirated and fixed with a cold methanol at 4 °C for 24 h. The samples were washed with PBS and stained with DAPI (Santa Cruz Biotechnology, Santa Cruz, CA, USA). The initial attachment of rCEnCs on the TCP, and films were investigated by fluorescence microscopy (Nikon Eclipse TE-2000U, Nikon, Tokyo, Japan), and the cell number was counted using the Image J program (*n* = 3).

### 2.7. Cell Viability

MTT (3-[4,-dimethylthiazol-2-yl]-2,5-diphenyltetrazoliumbromide;thiazolyl blue, Sigma-Aldrich, USA) assay was performed for the rCEnC viability on the SF and LPA/SF. The 100 cells/mm^2^ per well were seeded on TCP, SF, and LPA/SF and cultured in EGM-2. The rCEnCs were cultured for 1, 3, and 7 days, and the culture media was changed every 2 days. The samples were replaced with fresh culture medium with the addition of MTT solution (50 mg/mL in PBS) to make 10% MTT of medium volume and incubated under standard conditions (5% CO_2_, and 37 °C) for formazan crystal formation. After 4 h of incubation, the solution was aspirated and 1 mL of dimethyl sulfoxide (DMSO) was added to dissolve formazan crystal. Finally, the absorbance of the solution was evaluated at 570 nm using microplate reader (Synergy MX, Biotek, Vernusky, VT, USA) (*n* = 3).

### 2.8. mRNAs Expression

The rCEnCs seeded with the density of 100 cells/mm^2^ films and TCP were cultured for 3 and 5 days. Trizol (Invitrogen, Carlsbad, CA, USA) and chloroform (Sigma-Aldrich, USA) were used to extract mRNA and centrifuged at 12,000 rpm in 4 °C for 15 min. The supernatant was transferred to a 1.5 mL Eppendorf tube. Iso-propanol (Sigma-Aldrich, USA) was added and kept in 4 °C overnight. Isolated mRNA was dissolved in RNase-DNase free water (Gibco, USA). The gene markers of voltage-dependent anion-selective channel 2 (VDAC2), voltage-dependent anion-selective channel 3 (VDAC3), chloride channel protein 2 (CLCN2), and sodium/bicarbonate co-transporter (NBC1) were evaluated and normalized using β-actin a housekeeping gene. Gene expression was measured by electrophoresis on 1% (*w*/*v*) agarose gel containing Ethidium Bromide (EtBr, Sigma-Aldrich, USA). Images were obtained under a UV light (FluorChem FC2, Alpha Innotech, San Leandro, USA) at 360 nm.

### 2.9. Immunohistological Analysis

The expression of Na^+^/K^+^-ATPase and ZO-1 were measured to identify rCEnCs on SF and LPA/SF. The histological expression was evaluated after 3 days of culture. The rCEnCs were fixed with 4% formaldehyde (Sigma-Aldrich, USA) at 4 °C overnight and washed with PBS three times. A protein-blocking solution (DAKO, Glostrup, Denmark) was added for 15 min at room temperature to prevent non-specific binding. Fixed samples were incubated with the primary antibodies anti- Na^+^/K^+^-ATPase and anti-ZO-1 (1:200, Sata Crux Biotechnology, Dallas, TX, USA) at 4 °C for overnight. Alexa Fluor^®^594-conjugated AffiniPure Donkey Anti-Rabbit IgG (1:300, Jackson Immuno Research Laboratories, Inc., West Baltimore Pike West Grove, PA, USA) was used as a secondary antibody. The images were taken by confocal laser scanning microscope (LSM 510 META, Zeiss, Oberkochen, Germany) installed in the Center for University-Wide Research Facilities (CURF) at Chonbuk National University.

## 3. Results

### 3.1. Characterization of SF and LPA/SF

#### 3.1.1. ATR-FTIR Spectroscopy

**Figure 1:**
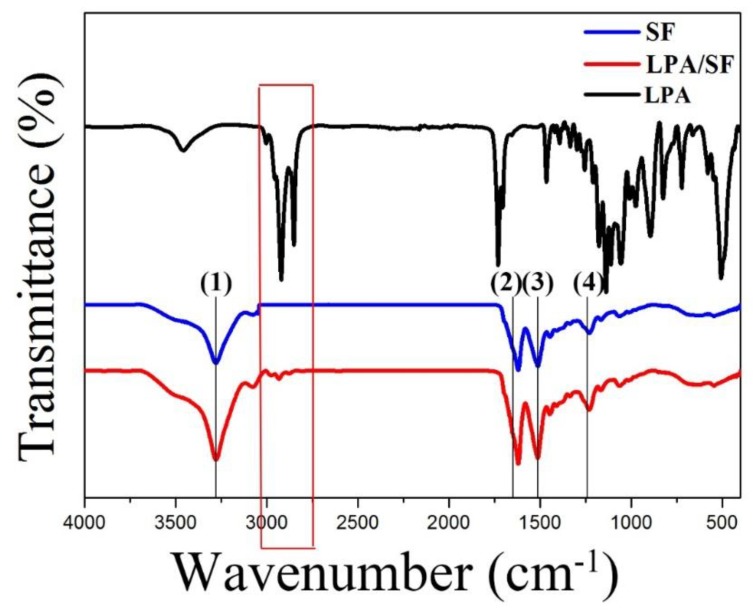
ATR-FTIR spectroscopy of SF and LPA/SF spectra wavelength rage of 4000 cm^−1^–400 cm^−1^.

The LPA, SF, and LPA/SF components and structure confirmation were analyzed by ATR-FTIR. The crystallized SF is shown in the 1700 cm^−1^–1200 cm^−1^ range. The β-sheet formation of the film is displayed by the three amide peaks. The C=O stretch, which is amide I (2), is shown at 1630 cm^−1^–1650 cm^−1^; amide II (3), which is N-H band, appears at 1520 cm^−1^–1540 cm^−1^ and C=N stretch the amide III (4) is shown at the range of 1230 cm^−1^–1270 cm^−1^. Also, the –OH peak (1) was shown at the range of 3000 cm^−1^–3650 cm^−1^. The result of LPA/SF shows deeper depth transmittance than the SF. The LPA/SF peak showed slightly different peak at the wavelength range of 3100 cm^−1^–2910 cm^−1^ compared to the SF (Figure 1).

#### 3.1.2. Transparency

**Figure 2:**
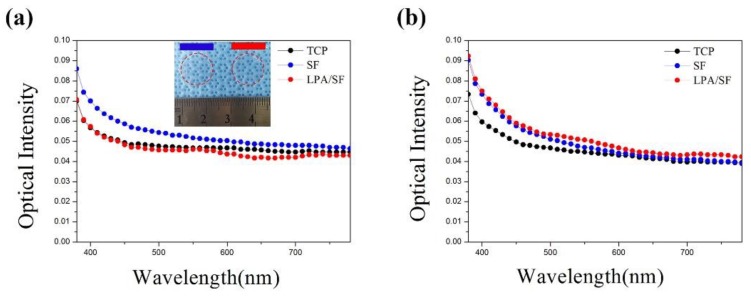
Gross image and transparency of SF (red) and LPA/SF (blue) at wavelength of 380 nm 780 nm (**a**) without cell culture and (**b**) with cell culture (*n* = 3).

The transparency of the films was studied by spectrophotometer at the wavelength range of 380 nm–780 nm. The gross image of SF and LPA/SF and the transparency of the fabricated films and TCP in the visible range is shown (Figure 2a,b). TCP, which is a commercially available material, was set as a positive control. The SF showed the highest transparency, but there was no significant difference between LPA/SF and TCP. The transparency of films with cell culture displayed LPA/SF slightly more transparently than TCP and the pristine SF.

#### 3.1.3. Hydrophilicity

**Figure 3:**
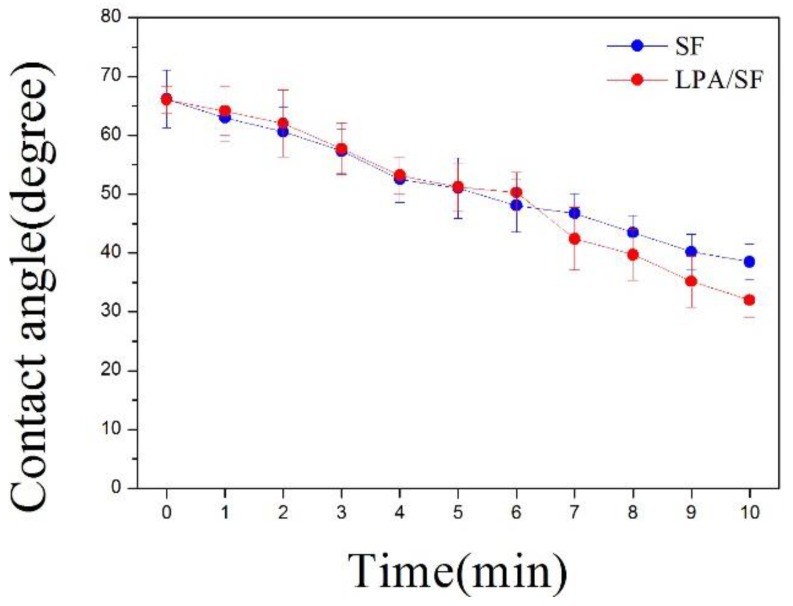
Contact angle of single water droplet (2 μL) on SF and 20 μM LPA/SF observed for 10 min (*n* = 5).

The contact angle of a water droplet on SF and LPA/SF was measured for 10 min. The difference of contact angle between SF and LPA/SF was not significant for 5 min. However, LPA/SF showed lower contact angle than the SF after 6 min (Figure 3).

#### 3.1.4. Surface Morphology and Roughness

**Figure 4:**
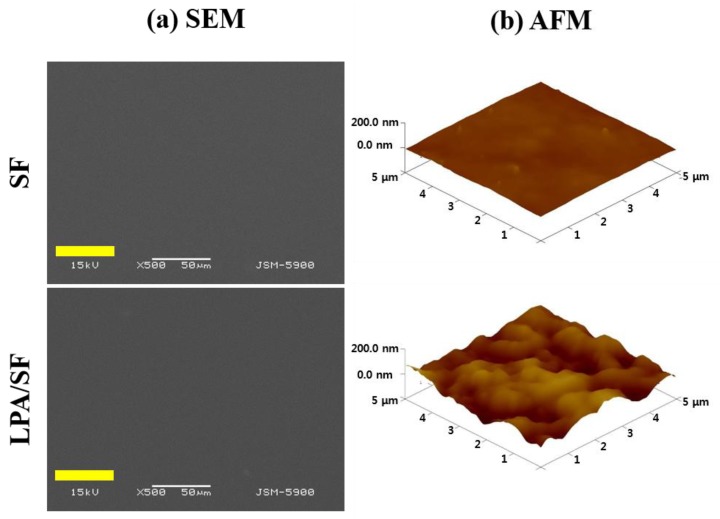
(**a**) FESEM and (**b**) AFM images of as-fabricated SF and LPA/SF.

The surface properties of the SF and LPA/SF were studied by FESEM and three-dimensional (3D) AFM images. There was no significant difference between each film on the FESEM image (Figure 4a). However, the topographic AFM images of bare SF and LPA/SF differed (Figure 4b). LPA/SF showed rougher surface when compared to the SF. 

### 3.2. In Vitro Study

#### 3.2.1. Monolayer Formation

**Figure 5:**
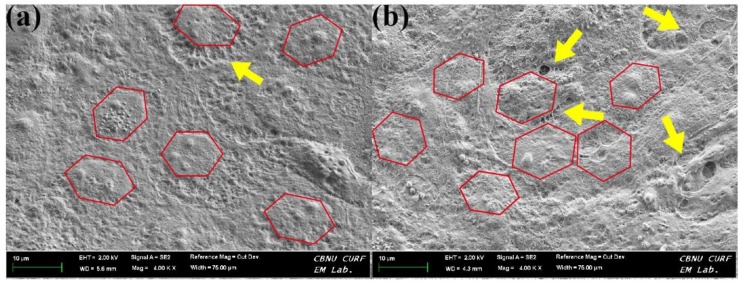
FESEM images of 5 days-cultured rCEnCs morphology on (**a**) SF and (**b**) LPA/SF.

The morphology of rCEnCs cultured on films was evaluated by FESEM. The morphology, cell density, and ECM secretion of rCEnCs were significantly different between SF and LPA/SF (Figure 5a,b). The hexagonal shape of cells (red line), which is the basic structure of the endothelial cells, was displayed in both films, but the ECM secretion between cells was significantly higher in LPA/SF (yellow arrow).

#### 3.2.2. Initial Attachment

**Figure 6:**
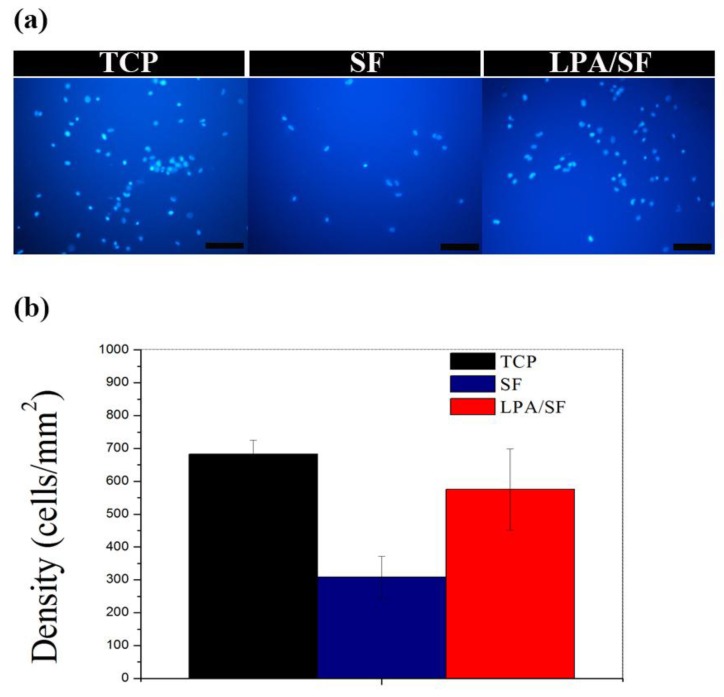
Initial attachment of rCEnCs on TCP, SF, and LPA/SF. (**a**) DAPI staining for initial attachment evaluation (scale bar 100 μm) and (**b**) initial attachment of rCEnCs on the films (*n* = 3).

The fluorescence image of TCP, SF and LPA/SF displayed different cell attachment. TCP showed the highest cells and LPA/SF presented analogous cell density to TCP (Figure 6a). The initial cell attachment on TCP the positive control was the highest (683.15 ± 42.66 cells/mm^2^). The LPA/SF displayed a similar level of TCP initial cell attachment (575.1 ± 123.34 cells/mm^2^). The SF cell density was the lowest (308.7 ± 63.12 cells/mm^2^) (Figure 6b).

#### 3.2.3. Cell Proliferation

**Figure 7:**
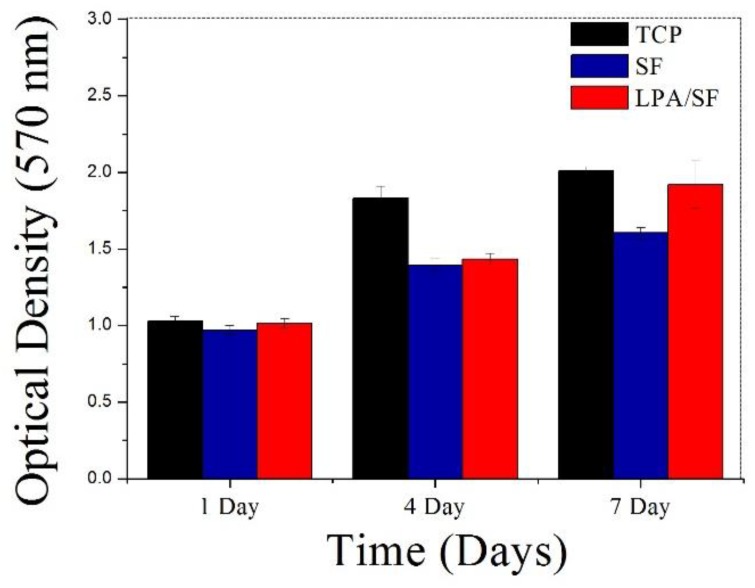
Proliferation assay of cell-cultured TCP, SF, and LPA/SF in EGM-2 (*n* = 3).

TCP showed the highest cell proliferation for 7 days. Compared to bare SF, LPA/SF was found to increase in cellular number. The LPA/SF displayed higher cell density for 7 days, which implies better rCEnCs growth than the SF (Figure 7).

#### 3.2.4. Specific mRNAs Expression

**Figure 8:**
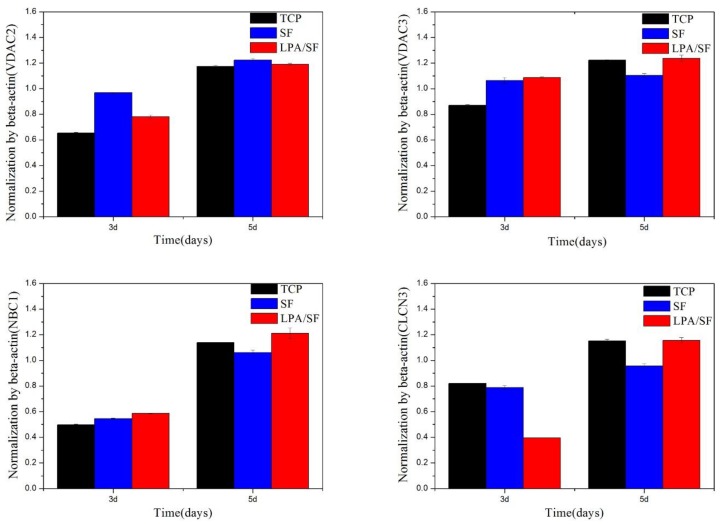
CEnCs-specific gene expressions of different films with cell culture by RT-PCR, normalized by β-actin (*n* = 3).

mRNAs expression was studied by RT-PCR using CEnCs-associated genes such as VDAC2, VDAC3, NBC1, and CLCN2. β-Actin housekeeping gene was used for normalization. TCP was set as a positive control. LPA/SF showed more enhanced gene expression for 5 days compared to SF alone (Figure 8).

#### 3.2.5. Immunohistological Evaluation

**Figure 9:**
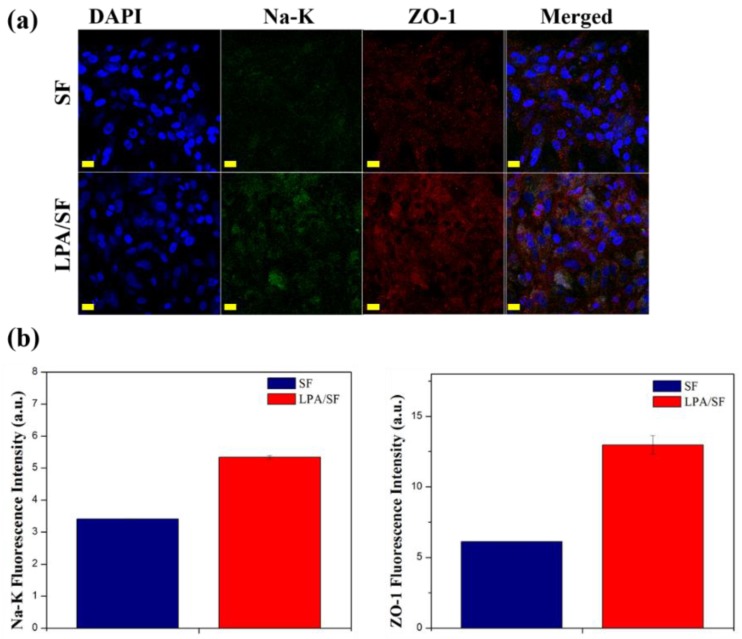
Immunofluorescence staining images of Na-K and ZO-1 of rCEnCs cultured on SF and LPA/SF (Scale bar 50 μm). (**a**) Fluorescence intensity of Na-K and ZO-1 analyzed by image J program (**b**).

The SF and LPA/SF cultured with rCEnCs were stained with Na^+^/K^+^-ATPase (Na-K), which is related to cornea transparency and tight junction protein ZO-1. Both films were properly expressed without any remarkable changes of rCEnCs morphology (Figure 9a). The LPA/SF showed higher fluorescence intensity, which was measured by the Image J program. (Figure 9b).

## 4. Discussion

The structure of SF and LPA/SF was confirmed by the ATR-FTIR spectroscopy. Both SF and LPA/SF displayed proper crystallized Silk fibroin [15]. The LPA/SF presented a small peak at the wavelength rage of 3100 cm^−1^–2910 cm^−1^, which is similar peak to the one shown in the LPA. Moreover, the LPA/SF exhibited deeper depth of the absorption band compared to SF. It is speculated that the increase in intensity may be due to the overlap of the LPA and silk fibroin peak. However, the concentration of the LPA in the SF was low that there was no particular difference.

Optically transparent SF and LPA SF were formed (Figure 2). The transparency is important in cornea tissue engineering to provide a clear vision in vivo and monitor cell behavior, process of healing, and check on any sign of infection [15,41]. Notably, the optical intensity of healthy human acellular corneal stroma is 0.1–0.13 at the wavelength of 380 nm–780 nm [2,42,43]. Figure 2 shows the as-fabricated films transparency at the wavelength range of 380 nm–780 nm. The pristine SF showed higher transparency than the LPA/SF. However, when films were cultured with rCEnCs, LPA/SF showed slightly higher transparency. This suggests that LPA/SF has the potential to be used as a corneal substitute.

Hydrophilicity property is important, because it effects cell attachment, proliferation, and migration on the substrate [28,29,35]. The measurement of contact angle is utilized to determine the hydrophilicity and applicability of the films. The hydrophilicity was evaluated at 0 min and the hydrophobicity was analyzed at 10 min to characterize the hydrophilicity/hydrophobicity. LPA-incorporated SF showed higher hydrophilicity than the bare SF (Figure 3). Hydrophilic film provides essential nutrients and prevents loss of body fluids [44,45,46].

The surface characteristics of film for CEnC regeneration is crucial for interaction between cells and proliferation [47]. The surface was identified by the FESEM and AFM. The FESEM result showed a smooth surface in both films (Figure 4a). However, the topography of the films was significantly different in AFM, which measures the surface roughness in nanoscale [48]. The roughness was significantly high in LPA/SF, whereas the SF presented relatively smooth surface (Figure 4b). It is reported that a rough surface provides greater cell attachment and a greater proliferation environment [48]. The result of the cell morphology on the films showed concomitant results of foregoing discussion. The films cultured with rCEnCs displayed monolayer on all films and presented hexagonal shape of rCEnCs. However, large amount of ECM release between rCEnCs in LPA/SF was exhibited (Figure 5b). Interaction between ECM and single layer of rCEnCs with the specific hexagonal shape regulates the hydration of corneal stroma and controls transparency of the cornea in the anterior chamber of eyeball [30,49,50].

The initial attachment to a graft, which defines affinity toward the specific substrate, is an important factor to consider in tissue engineering [28,32,35]. A high rate of initial attachment signifies that less time is required to reach the demanded confluency to the target graft and higher cell proliferation [15,43,45]. The evaluation of the initial cell attachment to the films showed that the LPA/SF was significantly higher than SF and similar rate to the positive control TCP (Figure 6a,b). The viability and proliferation of CEnCs is important for vision recovery when transplanted [34]. The density of CEnCs must reach around 500 cells/mm^2^, otherwise it cannot function properly and causes edema [15,51]. Both films increased significantly over 4 days compared to 1 day. However, the difference was observed after 7 days, at which point LPA/SF displayed significantly increased rate of proliferation as similar to the positive control TCP (Figure 7). It is predicted that LPA activated ligand of cells and led to cell proliferation [37]. Furthermore, the TCP is widely known as the best material for tissue culture of cell adhesion and proliferation [52]. However, TCP cannot be transplantable. The fact that LPA/SF results in analogous level of proliferation to the TCP suggests that LPA/SF is a beneficial material for CEnCs tissue engineering. This is because LPA/SF can be transplantable, and carries culture property similar to the TCP.

For further confirmation, specific mRNAs and functional protein expressions were obtained. VDAC2 and VDAC3 are known to regulate the interaction among cells, proteins, and small molecules [53]. CLCN2 controls pH, transports organic molecules, and effects cells migration, proliferation, and differentiation [34,54]. NBC1 is one of the NBC proteins and involves in absorption and secretion of cellular HCO_3_^−^ and also intracellular pH regulation [54]. LPA/SF cultured with rCEnCs showed more gene expression than SF on VDAC3, CLCN2, and NBC1 and slightly lower expression on VDAC2 on 7 days (Figure 8). Considering the fact that LPA is involved in epithelial growth, it is expected that LPA increases the favorable environment for cell growth and genes expression of SF [37]. Moreover, the LPA/SF displaying the similar level of gene expression to the positive control TCP is significant. This fact indicates that the LPA/SF can provide proper environment similar to the TCP. The protein expressions results showed that rCEnCs on all films were displayed properly without any remarkable changes in the cells morphology (Figure 9). Na-K is the typical CEnCs marker which is responsible in clearing water from the corneal stroma and providing transparency [2]. The LPA/SF exhibited higher expression of Na-K compared to the SF (Figure 9a,b). It is assumed that the LPA supported the SF to provide enhanced environment for rCEnCs functionality. The LPA/SF also showed higher expression level in ZO-1 which is a tight junction protein. This may be attributed to LPA considering the fact that LPA is known to stimulate cells growth and biological function [3,36,37,38,39,40]. In conclusion, LPA/SF provided similar condition to positive control TCP and higher CEnCs specific gene and protein expression. It is envisioned that LPA/SF can be a promising substrate for CEnCs delivery.

## 5. Conclusions

In this study, proper and enhanced film for corneal tissue regeneration was designed. Both SF and LPA/SF showed appropriate mechanical properties and enhanced biological properties in vitro. rCEnCs successfully adhered, proliferated, and expressed CEnCs specific genes and proteins on the both films. Further work is required to obtain an insight into how cells are modulated and respond to LPA/SF. Considering the overall results, it is expected that LPA/SF can be employed to enhance the clinical efficacy of the delivery of CEnCs to regenerate diseased or damaged CEnCs.
